# Development and Early Outcomes of the Watauga Compassionate Community Initiative, North Carolina

**DOI:** 10.13023/jah.0201.05

**Published:** 2020-01-26

**Authors:** Tim Marema, Erin Bouldin

**Affiliations:** Center for Rural Strategies, tim@ruralstrategies.org; Appalachian State University, bouldinel@appstate.edu

**Keywords:** Appalachia, adverse childhood experiences, abuse, health outcomes, community action

## Abstract

Addressing adverse childhood experiences has become a public health imperative, and communities across the United States are working to develop and implement programs and policies to both prevent childhood trauma and support adults who experienced trauma as children. Here we describe the development of the Watauga Compassionate Community Initiative (WCCI) in Watauga, County, North Carolina.

Each month in Watauga County, North Carolina, about 60 people meet in a county conference room. Among them are social workers and pastors, professors and healthcare providers, emergency responders and law enforcement officers. They come together because they are committed to preventing childhood trauma in their community and to helping adults who experienced trauma early in life to avoid or recover from negative outcomes like substance misuse, chronic disease, and incarceration. The group is called the Watauga Compassionate Community Initiative (WCCI) and its mission is “to promote health and resiliency in our community and to effectively prevent, recognize, and treat trauma by creating safe, stable, nurturing environments and relationships through education, advocacy and policy change.”^1^

The Watauga Compassionate Community Initiative grew out of a school social worker’s discovery of research about adverse childhood experiences (ACEs), which are traumatic experiences like abuse, witnessing violence in the household, or having a parent with mental illness or substance use disorder.^2^ In the late 1990s, data collected from more than 17,000 Kaiser Permanente enrollees in California showed that experiencing adverse childhood experiences was associated with an array of chronic health conditions and poor health outcomes including depression and suicide.^2^

Denise Presnell had practiced for years in schools across the county and had worked with children who experienced behavioral and academic problems at school and had challenging lives at home, but she had never heard of ACEs. Presnell says, “Learning about ACEs helped me understand why people make the choices they do. Their brains have been architectured in trauma and stress. They are in survival mode. Priorities for daily living are different when you are in survival mode.” Learning about ACEs and the research showing their relationship to a host of poor behavioral and health outcomes later in life was like discovering the answer to how to help students.

Presnell was already working with other youth-serving agencies and organizations in the county, including the local health department, the Western Youth Network, and the Children’s Council, led by Crystal Kelly. Together they discussed ACEs and agreed ACEs were a major upstream cause of poor outcomes in the county. Guided by the Centers for Disease Control and Prevention’s *Essentials for Childhood* Framework, the group decided to focus on raising community awareness and providing education about trauma and resiliency. (https://www.acesconnection.com/blog/essentials-for-childhood-framework)

In May 2017, they held a “State of the Child” forum with themes of childhood trauma and trauma-informed communities. Approximately 400 people attended, representing a range of individuals and organizations across Watauga County: law enforcement officers, healthcare providers, educators, youth group leaders, and parents. As part of the forum, participants were assigned to one of twenty concurrent brainstorming sessions that included people from different fields. They were asked, “In an ideal community, how could we more effectively prevent, recognize, and treat trauma?” Each brainstorming group considered what actions might be taken in 14 different sectors, including schools, youth and family service agencies, law enforcement, the faith community, and the medical community. The key themes were identified and shared during the final conference session and conference participants were invited to form committees that would use the brainstorming themes and next steps to continue the community’s efforts. Conference participants completed pre- and post-tests, which showed that ACEs knowledge and awareness increased as a result of the forum, and participant satisfaction was high.

Since that forum, the WCCI has developed as a collaborative network. WCCI includes a nine-person steering committee and five subcommittees: awareness, data, policy, prevention, and events. All members of the WCCI collective join one committee and help develop its agenda including action items that will achieve WCCI’s vision of Watauga County as a “relationship-driven, compassionate, and resilient community that is knowledgeable, inspired, and empowered to prevent harm, promote well-being, and heal from adversity.”^1^ Kelly describes the structure and process as follows: “As WCCI has grown we have organically developed a structure to organize our work. The committees allow for all members to have a role in healing our community and building protective factors for children to thrive. Our Leadership Team provides strategic oversight and aligns the committee work to the larger picture. We are constantly learning and changing and building it as we go.”

The Watauga Compassionate Community Initiative continues to use the *Essentials for Childhood* Framework as their organizing model, which includes four goals:

Goal 1: Raise awareness and commitment to promote safe, stable, nurturing relationships and environments and prevent child abuse and neglect;
Goal 2: Use data to inform actions;
Goal 3: Create the context for healthy children and families through norms change and programs; and
Goal 4: Create the context for healthy children and families through policies.^3^

During the 2 years of its existence, WCCI has made progress on each of these goals. For example, it has *raised awareness and commitment* by developing a business card with crisis resources for emergency responders, creating an informational presentation board housed at the local public library, speaking to numerous community groups, and hosting the annual “State of the Child” forum. In order to *use data to inform actions*, WCCI has created an interactive GIS resource map of the community, used state and local data like the Behavioral Risk Factor Surveillance System (BRFSS; https://www.cdc.gov/brfss/index.html) and Youth Risk Behavior Survey (YRBS; https://www.cdc.gov/healthyyouth/data/yrbs/index.htm) to measure ACEs, collected data on ACEs and resiliency from WCCI members, and tracked presentations and other community event attendance and outcomes. WCCI also has worked to *change norms and policies*. It has supported the development and expansion of trauma-informed schools with an aim of creating a compassionate school system across the county, collaborated with local law enforcement officers and judicial agencies to consider trauma history and incorporate treatment throughout the process from arrest through incarceration, worked to implement universal home visiting programs for families to provide support and early intervention as needed, and helped develop and deliver a course on trauma-informed care at Appalachian State University.

The work of Watauga Compassionate Community Initiative continues with monthly meetings and annual conferences. The third conference, held in May of 2019, asked “What’s STRONG in You?” and focused on resilience. Attendance totaled 585 people. In the future, the group plans to become more engaged in advocacy and to work directly with local elected officials to implement policies that will prevent ACEs and support recovery and resilience, develop stronger ties with healthcare providers, and expand its connection with the local university. Planning is underway for the fourth annual conference with the theme “Community is the Solution”. The attendance of the monthly meetings outgrew its meeting space and a new, bigger location was secured for meetings.

People outside the county are hearing about the work of WCCI, coming to attend the conferences, and calling to request information on starting similar initiatives in their communities. WCCI also aims to be sustainable, including financial sustainability in the form of grants and external funding and administrative sustainability in the form of distributing responsibilities across more members to prevent burnout. To date, the group has applied for funding from the Robert Wood Johnson Foundation, the Blue Cross BlueShield of North Carolina Foundation, and the Mountain Area Health Education Center (MAHEC). Recently, WCCI was selected as one of three communities in the state to receive funding to participate in the first cohort of the North Carolina ACE Learning Collaborative.

Addressing ACEs has become a public health imperative,^4^,^5^ and communities across the U.S. are working to develop and implement programs and policies to both prevent childhood trauma and support adults who experienced trauma as children. ACEs Connection (https://www.acesconnection.com/) offers networking opportunities among its 35,000+ members and resources (e.g., presentation templates, webinars, and sample policies/legislation) for communities interested in developing an initiative like WCCI. Washington state was a pioneer in addressing ACEs at the state and local level and their experience may serve as a helpful roadmap for other communities interested in this work.^6^ ACEs are common both within and beyond Appalachia and communities across the Appalachian region can take steps to recognize and reduce childhood trauma and foster resilience among those who experienced trauma when they were children.
